# MiR-608 overexpression in idiopathic pulmonary fibrosis (IPF)

**DOI:** 10.1186/s12890-020-01377-3

**Published:** 2021-01-05

**Authors:** Gali Epstein Shochet, Lilach Israeli-Shani, Isabelle Kains, Ori Wand, David Shitrit

**Affiliations:** 1 Pulmonary Department, Meir Medical Center, 59 Tchernichovsky St., 44281 Kfar Saba, Israel; 2 Sackler Faculty of Medicine, Tel Aviv University, Tel Aviv, Israel

**Keywords:** microRNA, IPF, ILD, SNP, CdC42

## Abstract

**Background:**

Idiopathic pulmonary fibrosis (IPF) is a chronic progressive disease that causes scarring of the lungs. The disease is associated with the usual interstitial pneumonia pattern, which was not yet fully recapitulated by an animal model. Therefore, the disease is considered ‘human specific’. miRNA-608 is a primate specific miRNA with many potential targets, such CdC42 and Interlukin-6 (IL-6) that were previously implicated in IPF pathology.

**Objective:**

To test miR-608 expression and its targets in IPF patient samples.

**Methods:**

RNA was extracted from Formalin fixed paraffin embedded tissue sections (N = 18). miRNA-608 and Cdc42 and IL-6 levels were analyzed by qPCR. Acetylcholinesterase (AChE) is another target of miRNA-608. Its’ rs17228616 allele has a single-nucleotide polymorphism causing weakened miR-608 interaction (C2098A). Thus, DNA was extracted from whole blood samples from 56 subjects with fibrosing interstitial lung disease and this region was sequenced for assessment of rs17228616 allele polymorphism.

**Results:**

miR-608 is significantly overexpressed in IPF samples in comparison with controls (*p* < 0.05). Cdc42 and IL-6 levels were lower in the IPF patient samples compared with control samples (*p* < 0.001 and *p* < 0.05, respectively). The frequency of the rs17228616 minor A-allele was 17/56 (30.4%) with all patients being heterozygous. This result is significant vs. the published Israeli cohort of healthy individuals, which reported 17% prevalence of this allele in healthy control volunteers (*p* = 0.01, OR = 2.1, CI 95% [1.19–3.9]).

**Conclusion:**

miR-608 is overexpressed in IPF patients. While the exact mechanism remains to be discovered, it could potentially promote fibrotic disease.

## Introduction

Idiopathic pulmonary fibrosis (IPF) is a chronic disease that causes scarring of the lungs. The disease is characterized by progressive worsening of dyspnoea and decline in lung function. Many patients with IPF have sub-clinical or clear co-morbid conditions including pulmonary hypertension, gastroesophageal reflux, obstructive sleep apnoea, obesity, emphysema, as well as depression and anxiety [1, 2].

The disease is limited to the lungs, and is associated with the histopathologic and/or radiologic pattern of usual interstitial pneumonia (UIP). So far, no animal model fully recapitulated the UIP pattern [3] and the disease is considered ‘human specific’.

Micro-RNAs (miRNA) are a non-coding RNA sequences, of about 22 nucleotides long that have complementary sequences to particular regions of the mRNA, often in the 3′UTR, through which regulation occurs. Regulation generally proceeds via either mRNA degradation or inhibition, leading to gene silencing, with the degree of inhibition highly depending on the degree of complementarity between the miRNA and its corresponding mRNA target [4, 5].

Single-nucleotide polymorphisms (SNPs) are strongly associated with susceptibility to various diseases, including IPF [6–8]. SNPs within miRNA sequences may therefore change properties of the resulting inhibition by altering degree of complementarity [9, 10].

miRNA-608 (miR-608, hsa-mir-608) is a long (25 nucleotides) primate specific miRNA. It has many potential targets, such as Rho GTPase CdC42 and Interlukin-6 (IL-6), bearing much experimental evidence [11, 12]. It was previously shown that the miR-608 has differential affinity to the AchE sequence due to C to A change (C2098A) in its 3′-untranslated region (e.g. the minor ‘A-allele’ and the major ‘C-allele’, respectively), indicating weakened A-allele AChE–miR-608 interaction [11]. The impaired interaction of the A-allele of AChE with miR-608 predicted weakened AChE suppression, resulting in elevated levels of free miR-608 molecules to suppress other targets with tight miR-608 binding sites, such as Cdc42 and IL6, which were already shown to be involved in IPF progression [13, 14]. In this study, we tested miR-608 expression and its targets in human IPF patient samples.

## Materials and methods

### RNA purification and quantitative PCR (qPCR)

Total RNA (including miRNA) was extracted from Formalin fixed paraffin embedded (FFPE) tissue sections taken from 18 IPF patients and 8 control samples, using the miRNeasy FFPE kit (Qaigen, USA) according to the manufacturer’s instructions.

mRNA was converted to cDNA using the reverse transcription kit (Applied Biosystems, UK). Reactions were performed with SYBR Green PCR master mix (Applied Biosystems, UK). Primer sequences (purchased from Hylabs, Israel) are listed in Table 1. 18S served as the reference housekeeping gene. Primers were normalized by specific cDNA standard curves obtained from known amounts of cDNA.

**Table 1:** List of primers

	Forward (5′–3′)	Reverse (5′–3′)
CDC42 variant 1 (NM_001791)	CTGTCAAGTATGTGTGGAGTGTTCTG	CTCTTCTTCGGTTCTGGAGGCT
CDC42 variant 2 (NM_044472)	TGCACTTACACAGAAAGGCC	CTTCTTCGGTTCTGGAGGCT
18S	AGGAATTGACGGAAGGGCAC	GGACATCTAAGGGCATCACA
IL-6	GGTACATCCTCGACGGCATCT	GTGCCTCTTTGCTGCTTTCAC

miRNA was converted into cDNA using the qScript microRNA cDNA synthesis kit (Quanta Biosciences, USA) and was then amplified using Perfecta Universal PCR primer kit (Quanta Bioscience, USA). qPCR was performed with Perfecta SYBR Green using specific primers for mir-has-608 (Quanta bioscience, USA). The reference genes (Snord 44 and RNV6) primers were supplied with the kit (sequence not available).

### Human blood samples

Whole blood samples were collected from 56 subjects with fibrosing interstitial lung disease (ILD). DNA was purified from these samples using QaiSymphony (Qaigen). Their demographic data, as well as disease progression and final diagnosis were also recorded.

### Gene sequencing

DNA was amplified using PCRBIO HS Taq master mix (PCR Biosystems) with specific primers for the AchE: Forward 5′-CGCTGGAGCTCCTACATGGT-3′ and Reverse 5′-ATAGACTCGGCCCCGTGAT-3′. Products were purified using FastAP Thermosensitive Alkaline Phosphatase (Thermo-Fisher scientific). Then, sequencing was performed using BigDye™ Terminator v3.1 Cycle Sequencing Kit (Thermo-Fisher Scientific) according to manufacturer’s instructions. The target sequence was analysed by 3130 Genetic analyser (Applied Biosystems) for determining A/C allele single nucleotide polymorphism (SNP) at rs17228616.

### Statistical analysis

Statistical analysis was done using GraphPad Prism version 7.00 for Windows (GraphPad Software, La Jolla California USA, www.graphpad.com). ANOVA was performed to compare differences between multiple cohorts. Student’s *t* tests were employed to analyze differences between two groups. Frequencies were calculated using a Chi-square test. An effect was considered significant when the *p* value was < 0.05. All experiments were repeated at least three times.

### Ethical approval

This study was approved by the local Ethics Committee (MMC-18-18). Informed consent was obtained from all subjects.

## Results

### MiR-608 is overexpressed in lung tissue samples from IPF patients

**Fig. 1:**
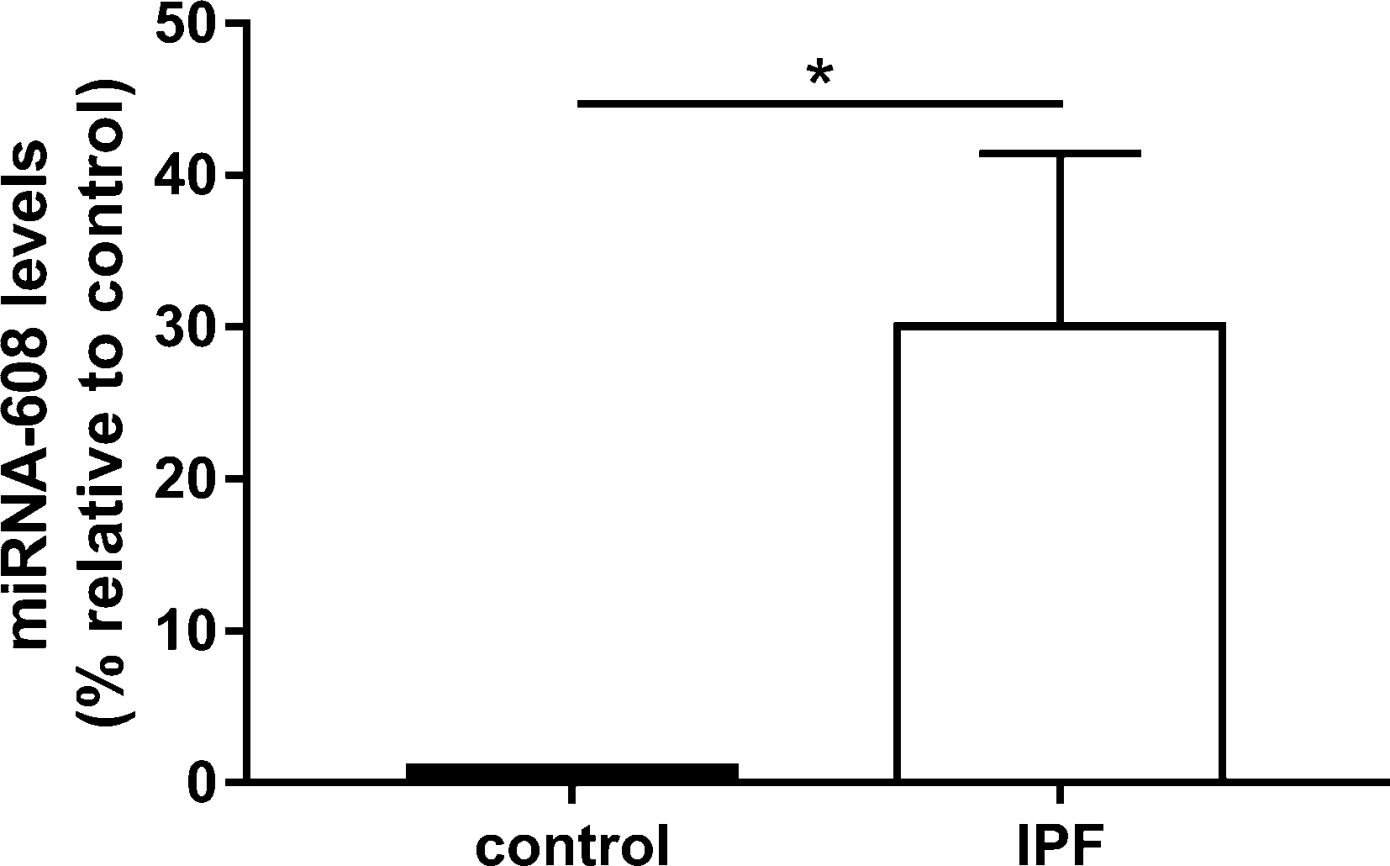
miR-608 is overexpressed in lung tissue samples from IPF patients. RNA was extracted from IPF and non-IPF (control) FFPE samples. miRNA-608 levels were evaluated by qPCR. Figure shows mean ± SEM. ***p ≤ 0.001, Student’s paired *t* test (n = 18)

The expression of miR-608 in was evaluated in IPF FFPE patient samples (n = 18) versus histologically confirmed control tissue samples (n = 8). The IPF group consisted of 50% males, with the average age of 65.5 ± 8.5 years. The control group also consisted of 50% males, with the average age of 59.3 ± 18 years. There were no significant differences between the groups regarding comorbidities (other than ILD). Interestingly, a significant overexpression of miR-608 was found in IPF patient samples, in comparison with controls (*p* < 0.05, Fig. 1). Using the miRDB search (http://mirdb.org/cgi-bin/search.cgi), 975 possible targets for miR-608 were identified. These results were then subjected to WEB-based Gene set analysis toolkit (WebGestalt, http://www.webgestalt.org). We analysed the overrepresentation analysis (ORA) for disease (OMIM) functional database. Interestingly, IPF was the most significantly over-represented disease, with an enrichment ratio of 78 (PULMONARY FIBROSIS, IDIOPATHIC, gene set 178500, FDR = 0.00025).

### Cdc42 levels are significantly reduced in IPF patient samples

**Fig. 2:**
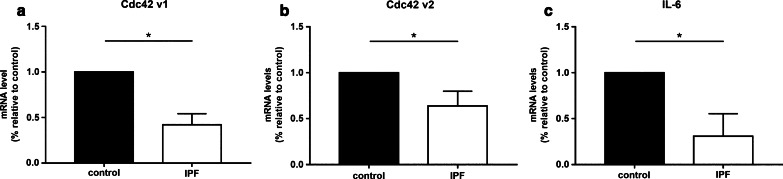
miR-608 target levels are significantly reduced in IPF patient samples. RNA was extracted from IPF and non-IPF (control) FFPE samples. The two variants of Cdc42 (**a, b**) and IL-6 (**c**) mRNA levels were evaluated by qPCR. Figures show mean ± SEM, *p ≤ 0.5, Student’s paired *t* test (n = 15)

RhoGTPase Cdc42 is an established target of miR-608 [11]. Moreover, it was recently implicated in lung fibrosis [13]. Thus, we measured the expression level of Cdc42 in IPF patient samples, in comparison to controls. Both Cdc42 transcript variant levels (v1 NM_001791 and v2 NM_044472) were significantly lower in samples from IPF patients compared to the control samples (*p* < 0.001, Fig. 2a, b).

### IL-6 levels are downregulated in IPF patient samples

IL-6 is well-known cytokine in IPF and another validated target of miR-608 [11]. qPCR was performed to measure the expression of IL-6 in IPF patients compared with healthy individuals. In fact, IL-6 levels were lower in the IPF patient FFPE samples compared with controls (*p* < 0.05, Fig. 2c). This result is in key with the hypothesis stating an inhibitory relationship between miR-608 and its targets.

### The C2098A substitution (minor rs17228616 allele) at the AChE sequence is more prevalent in IPF patients

Since both IL-6 and Cdc42 were downregulated in the FFPE samples from patients with IPF, we hypothesized that the presence of the minor A-allele (C2098A) could be higher in subjects with IPF. Thus, 56 patients were recruited and their DNA was sequenced for the A/C allele. Patient characteristics are listed in Table 2. 62.5% were male with the average age of 65.82 ± 12.

**Table 2:** Patient characteristics

Parameter	A-allele n = 17	C-allele n = 39	*p* value
Age	62 ± 14	62.4 ± 11	0.9
Gender (%male)	12 (70%)	23 (58.9%)	0.41
Smoker	13 (76.4%)	20 (51.2%)	0.08
Rapidly progressing disease^a^	6 (35.2%)	8 (20.5%)	0.24
FVC %	59 ± 14.5	70 ± 19	0.02
DLCO %	46.2 ± 17	48.6 ± 17	0.66
BMI	27.6 ± 5	28.4 ± 4.5	0.61
IHD	5 (29.4%)	7 (17.9%)	0.34
CHF	1 (5.9%)	2 (5.1%)	0.9
Diabetes	7 (41.2%)	16 (41%)	1
Anxiety	1 (5.9%)	6 (15.4%)	0.3
Hypertension	6 (35.2%)	11 (28.2%)	0.64
Osteoporosis	4 (23.5%)	7 (17.9%)	0.63
Malignancy	1 (5.9%)	5 (12.8%)	0.44

*BMI* body mass index, *CHF* Congestive Heart Failure, *DLCO* diffusing capacity for carbon monoxide, *FVC* forced vital capacity, *IHD* Ischemic heart disease

^a^A fall in %FVC of over 10% per year was defined as rapidly progressing

**Fig. 3:**
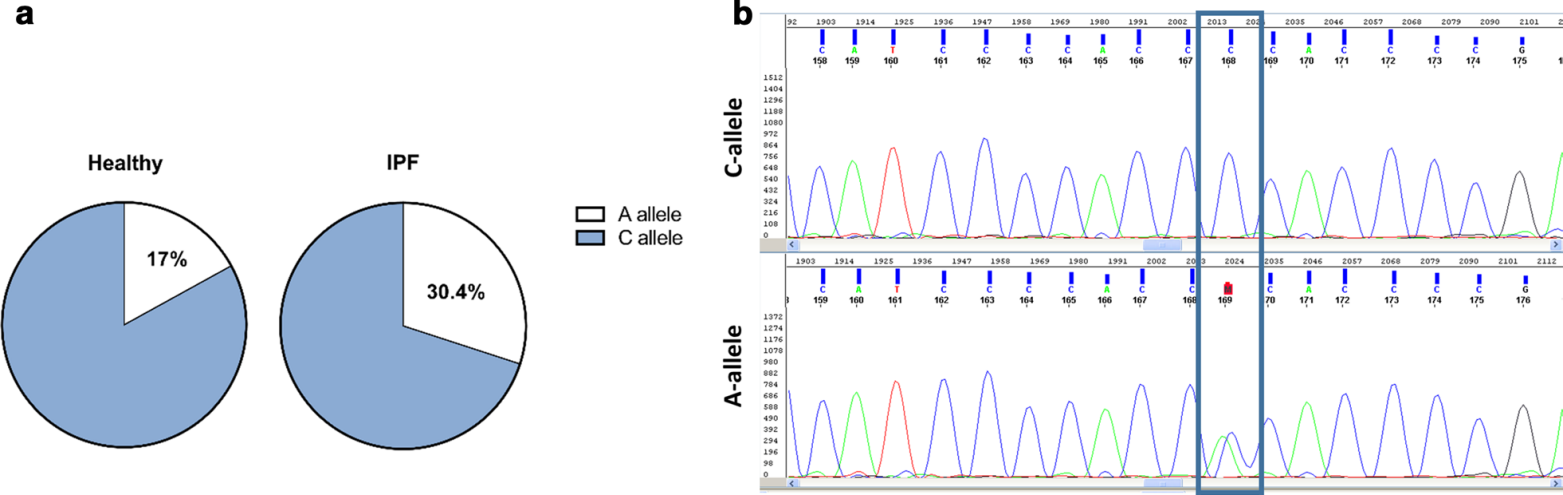
The C2098A substitution (minor rs17228616 allele) at the AChE sequence is more prevalent in IPF patients. DNA was extracted from 56 subjects with progressive fibrosing ILD. **AChE** miR-609 target sequence was analysed for determining A/C allele rs17228616 SNP (**a**). **b** is a representative image of sequencing output showing C (top panel) and A heterozygous allele (bottom panel)

Of these subjects, the frequency of the A-allele was 17/56 (30.4%) with all patients being heterozygous for the minor A-allele (Fig. 3). This result is significant vs. the published Israeli cohort of healthy individuals, which reported 17% prevalence of this allele in healthy control volunteers (*p* = 0.01, OR = 2.1, CI 95% [1.19–3.9]).

Of them, 64.5% were diagnosed with IPF and the rest with other types of ILD [e.g. NSIP (14.5%), silicosis (3.6%), hypersensitivity pneumonitis (HP, 3.6%), sarcoidosis (1.8%) and the rest were determined as unclassifiable pulmonary fibrosis ILD (12%)]. In addition, we followed up these patients’ disease progression to determine whether they are rapidly progressing, as previously defined by the annual decline in FVC% [15–18]. We found that of the minor allele population, more patients presented with a rapid progressing disease (35.4% vs. 20%, *p* = 0.24), yet this result did not reach significance.

When comparing between the A to the C allele groups' lung function tests at the time of diagnosis, a significantly reduced FVC at diagnosis for the A group was observed (Table 2, *p* = 0.02).

## Discussion

Numerous pathways were implicated and major progress has been made towards understanding IPF etiology [19]. However, as recently stated by McDonough et al. [20], since the UIP pattern cannot be replicated in animal models, we have no information about what regulates progression of IPF in the human lung. Thus, in this work we focused on the primate specific miRNA that was previously implicated in aging related diseases. We found that miR-608 was significantly upregulated in IPF tissue samples. In addition, we found that the minor rs17228616 allele was more abundant in IPF patients than in the general population.

miR-608 is located on human chromosome 10q24.31. Although not aforementioned in IPF [21], current studies in tumors indicate that miR-608 affects cell proliferation, invasion, migration and apoptosis [22, 23]. Although the expression level of miR-608 was found to be downregulated in several types of cancer [10, 24–27], these studies did not take into account the SNPs that can significantly affect miRNA stability and function [9].

The major SNP of miR-608 mentioned in cancer is the rs4919510 variant G allele. This SNP was suggested to affect the expression of mature miR-608, as well as that of the proinflammatory cytokines TNF-α, IL-6, and IL-1β. Nevertheless, there are conflicting results for the association between the presence of miR-608 rs4919510 and susceptibility to tumors [10, 28–30]. Although IPF and lung cancer are sometimes seen in the same patients [31], since we observed an upregulation in the miR-608, rs4919510 was not studied and our focus was shifted to other directions.

To date, limited targets of miR-608 have been confirmed in-vitro, and were mostly performed in tumor cell lines [32]. Our study focused on two targets that were previously implicated in IPF, IL-6 and Cdc42 [13, 14]. Both targets were found to be down-regulated in IPF patient samples. Supporting these results, our recent work, showed that the IL-6R protein level is also reduced in tissue samples taken from IPF patient biopsies [14]. A recent study by Wu et al. [13], suggested that Cdc42 is an important post-transcriptional regulator that may play a significant role in the process of inflammation. They found that AT2 cells that lack Cdc42 are not able to differentiate into AT1 cells and, thus, cannot regenerate new alveoli following lung injury. Although IPF is not considered to be an inflammatory disease per se, pro-inflammatory factors, such as IL-6, TNF-alpha and IFN-y were shown to contribute to disease progression [14, 33].

Our work was inspired by a group of researchers who studied miR-608 in the context of anxiety [11]. Several studies reported that symptoms of depression and anxiety are common in patients with IPF. Such studies indicated prevalence of depression ranges from 24.3 to 49.2%, while that of anxiety reaching 60% in patients with IPF and other ILDs [34–38]. Although a causality of anxiety or depression could be expected, it is possible to assume a genetic predisposition as well. However, the presence of anxiety was not fully addressed in this work, as it relied on electronic patient records and therefore is most likely underestimated or under-recorded [39]. For this issue to be addressed, there is a need for questionnaires, which were not done in this study.

The altered interaction of miR-608 with AChE and the resulting changes, which give rise to a higher ratio of suppression by miR-608 of its other targets, including CdC42 and IL-6, correlate to our results. Hanin et al. showed that young, healthy volunteers with the minor rs17228616 allele showed elevated blood pressure and reduced cortisol, predicting risk of aging-related diseases, such as IPF. Our cohort of patients with progressive fibrosing ILDs, mostly IPF, was shown to include significantly higher prevalence of minor A-allele in comparison to the healthy cohort presented by this group. Since both populations were from Israel, we can also assume similar genetic backgrounds. These results require further investigation in a large cohort to determine the polymorphism in this patient population.

In conclusion, although the number of patients was limited, a significant effect was reached. We found that miR-608 is overexpressed in IPF patients, and that this population includes 30% of a specific SNP in AChE that was previously implicated as relevant to aging related diseases. These findings require further research in a large study cohort.

## Data Availability

The datasets generated and/or analyzed during the current study are not publicly available due to patient confidentiality, but are available from the corresponding author on reasonable request.

## Abbreviations

AchE: Acetylcholine esterase
BMI: Body mass index
CHF: Congestive Heart Failure
DLCO: Diffusing capacity for carbon monoxide
FFPE: Formalin fixed paraffin embedded
FVC: Forced vital capacity
IHD: Ischemic heart disease
IL-6: Interlukin-6
ILD: Interstitial lung disease
IPF: Idiopathic pulmonary fibrosis
ORA: Overrepresentation analysis
SNP: Single-nucleotide polymorphisms
UIP: Usual interstitial pneumonia
